# Psychometric Evaluation of the Serbian MacNew Heart Disease Health-Related Quality of Life Questionnaire Before and After Elective Coronary Artery Bypass Grafting

**DOI:** 10.3390/epidemiologia7050144

**Published:** 2026-10-15

**Authors:** Ivan Nesic, Olufemi O. Oyewole, Petar Vukovic, Marko Kaitovic, Petar Tomic, Milica Ludoski, Djordje Krstic, Aleksandar Nenadovic, Aleksandar Milutinovic, Jelena Dotlic, Aleksandra Sljivic, Tatjana Gazibara

**Affiliations:** 1 Clinic of Cardiac Surgery, Institute for Cardiovascular Diseases “Dedinje”, 11000 Belgrade, Serbia; dr.inesic@gmail.com (I.N.); petarviuk02@gmail.com (P.V.); markokaitovic1@gmail.com (M.K.); ptomic997@gmail.com (P.T.); ludoski85@gmail.com (M.L.); krsticdjordje88@gmail.com (D.K.); 2 Faculty of Medicine, University of Belgrade, 11000 Belgrade, Serbia; drenadot@gmail.com; 3 Department of Physiotherapy, Olabisi Onabanjo University Teaching Hospital, Sagamu 121001, Ogun State, Nigeria; oyewoleye@gmail.com; 4 School of Health Sciences, University of KwaZulu-Natal, Durban 4001, South Africa; 5 Clinic for Anesthesia and Intensive Care, Institute for Cardiovascular Diseases “Dedinje”, 11000 Belgrade, Serbia; aleksandarnenadovic@yahoo.com; 6 Heart Surgery Department, University Children’s Hospital Tirsova, 11000 Belgrade, Serbia; aleksandarm1982@yahoo.com; 7 Clinic for Obstetrics and Gynecology, University Clinical Center of Serbia, 11000 Belgrade, Serbia; 8 Department of Cardiology, University Clinical Hospital Center “Dr. Dragisa Misovic—Dedinje”, 11000 Belgrade, Serbia; sljivic0809@gmail.com; 9 Institute of Epidemiology, Faculty of Medicine, University of Belgrade, 11000 Belgrade, Serbia

**Keywords:** MacNew heart disease health-related quality of life questionnaire, coronary artery disease, coronary artery bypass grafting, validation

## Abstract

**Background/Objectives**: To evaluate the psychometric properties of the MacNew Heart Disease Health-Related Quality of Life Questionnaire (MacNew HRQL) in patients who underwent elective coronary artery bypass grafting (CABG) in Serbia. **Methods**: Recruitment of participants was organized at the Institute for Cardiovascular Diseases “Dedinje”. The MacNew HRQL was administered prior to having CABG, and 1, 6 and 12 months after CABG. The scale was compared to the Short Form-36 (SF-36) and Depression, Anxiety and Stress Scale-21 (DASS-21). Clinical data were obtained from medical records. **Results**: A total of 192 patients had complete follow-up data (80.2% male, 64.1 ± 7.6 years old). The Cronbach’s alpha and McDonald’s omega coefficients were ≥0.80. The original three-factor structure was supported by indices on the confirmatory factor analysis using the Unweighted Least Squares estimator (ULS) at 6 and 12 months post-CABG, but not at baseline and 1 month after CABG. Longitudinal measurement invariance suggested that models were not supported. The MacNew HRQL global score correlated with clinical parameters at all four time points. All MacNew HRQL domains correlated with mental and physical scores of the SF-36 and with depression, anxiety and stress scores. **Conclusions**: The MacNew HRQL in Serbian had good internal consistency and construct validity. The three-factor structure was most favorable under the ULS at 6 and 12 months post-CABG. As the measurement invariance was not supported, changes in scores over time cannot be interpreted unequivocally as changes in the same underlying constructs. The scores right before and after CABG should be interpreted with caution.

## 1. Introduction

Cardiovascular diseases place a substantial burden on health systems worldwide [1]. Coronary artery disease (CAD) remains a leading cause of morbidity, disability, and mortality and requires continued efforts to optimize secondary and tertiary prevention [2]. Current therapeutic approaches for CAD include both non-invasive and invasive treatment [3]. However, coronary artery bypass grafting (CABG) represents one of the most effective therapeutic options for patients with advanced CAD [3].

The goal of CABG is to alleviate CAD symptoms, improve survival, and enhance patient’s functional capacity [4]. As contemporary treatment strategies emphasize patient-centered outcomes, the assessment of health-related quality of life (HRQL) has become an essential component of cardiovascular care and outcome evaluation in cardiac surgery [5]. Bearing in mind the impact of CAD on the physical, emotional and social aspects of daily life, the instruments examining HRQL should, therefore, extend beyond measures of physical health and include broader dimensions of self-perceived health.

While there are various questionnaires available to assess HRQL, the disease-specific instrument, the MacNew Heart Disease Health-Related Quality of Life Questionnaire (MacNew HRQL), evaluates physical, emotional, and social aspects of HRQL in patients with CAD [6]. As such, it provides a comprehensive assessment of patients’ self-rated well-being and can be used longitudinally to evaluate recovery following cardiac events or interventions [7,8]. Over the past two decades, the MacNew HRQL has been widely applied in international research, with studies conducted across diverse cultural and clinical settings consistently reporting good psychometric properties [9].

In the Republic of Serbia, where cardiovascular diseases remain the leading cause of morbidity and mortality [10], the use of validated questionnaires is particularly needed to evaluate the outcomes of CAD-specific treatments, including CABG. Evaluation of psychometric characteristics of such questionnaires would not only facilitate clinical monitoring and outcome assessment, but also enable meaningful comparisons of other populations. Although previous studies compared MacNew HRQL scores before and after CABG [7,11], longitudinal evaluation of psychometric characteristics across multiple time points remains limited. In longitudinal studies with multiple measurements, it is also possible to evaluate the structural validity of the questionnaire at different points in time, longitudinal measurement invariance and postoperative score change, which add valuable new pieces of information to the body of literature on the MacNew HRQL questionnaire.

To address this gap, the present study aimed to evaluate psychometric properties of the MacNew HRQL in a cohort of Serbian-speaking patients with CAD who underwent CABG.

## 2. Material and Methods

### 2.1. Ethics Approval

This study was approved by the Ethics Committee of the Institute for Cardiovascular Diseases “Dedinje” (approval no. 6038). All patients signed an informed consent form for participation. The study was conducted in line with the principles outlined in the Declaration of Helsinki.

### 2.2. Study Design

A prospective cohort study was carried out on 210 patients who underwent CABG and were followed for 12 months. Recruitment of the study cohort was performed from 1 July 2021 to 31 December 2023. The follow-up was completed on 31 December 2024.

#### Participants

A total of 210 consecutive patients with CAD scheduled for CABG were recruited at the Department of Preoperative Patient Preparation, Clinic for Cardiac Surgery, Institute for Cardiovascular Diseases “Dedinje”, a high-volume center for cardiac surgery in Belgrade, Serbia. The inclusion criteria were: age ≥ 18 years, having an elective CABG procedure in accordance with the Guidelines for surgical myocardial revascularization established by the European Society of Cardiology and the European Association of Cardiothoracic Surgery, being fluent in Serbian language and signing a consent form for participation. The exclusion criteria were: (1) needing surgical intervention on valves, combined cardiac surgical procedures, and/or cardiac and vascular surgical interventions; (2) having an urgent CABG procedure (due to acute coronary syndrome); (3) having a repeat cardiac surgical intervention; (4) needing hemodialysis; (5) having any neurological impairment; (6) having confirmed psychiatric illnesses including the intake of psychoactive drugs; (7) having cognitive impairment, dementia, or congenital intellectual disability; (8) partial or complete amputation of a limb; (9) advanced peripheral vascular disease; and (10) being diagnosed with or treated for malignancies. These criteria were set to minimize confounding by indication, as all of these illnesses and conditions affect HRQL perception.

The sample size was calculated with the following parameters in mind: power of 0.8, probability level of 0.05, Root Mean Square Error of Approximation (RMSEA) of <0.08 and 26 degrees of freedom for each item in the questionnaire. The minimum sample size was 140 participants.

### 2.3. Data Collection

Electronic medical records and questionnaires were used to collect data. The following data were retrieved from electronic medical records: sex, age, anthropometric measurements (height, weight), body mass index, left ventricle ejection fraction (LVEF), New York Heart Association (NYHA) classification, Canadian Cardiovascular Society (CCS) Angina Grading Scale, presence of dyslipidemia, diabetes mellitus, smoking, and family history of cardiovascular diseases.

The following questionnaires were administered: the MacNew Heart Disease Health-Related Quality of Life Questionnaire (MacNew HRQL), the Short-Form 36 (SF-36) and the Depression, Anxiety and Stress Scale-21 (DASS-21).

The MacNew HRQL is a specific questionnaire measuring health-related quality of life in people with heart disease. The permission to use the questionnaire was granted by the scale authors [12,13], and they also provided the Serbian version of the MacNew HRQL. It consists of 27 items related to the participants’ feelings about their physical, emotional and social health over the past two weeks. The last item in the questionnaire, item #27, refers to sexual functioning, i.e., the level to which participants’ heart disease limits them in sexual activity. This is the only item with a response option of “not applicable to me”. The authors of the MacNew HRQL [6] highlighted that this item may yield many missing responses, which do not contribute to the domain score or the global score. This was the case in our study—on the preoperative assessment, there were 14.6% of “not applicable to me” responses, and this proportion increased after CABG—37% at month 1, 41.1% at month 6 and 42.7% at month 12. Bearing in mind these large proportions of non-applicable responses and, hence, the missing data relative to scores on other items could not be meaningfully interpreted. Therefore, the Serbian version of the MacNew HRQL had 26 items.

Responses are graded on a seven-point Likert-type scale from 1 to 7, where a value of 1 indicates the worst possible health and 7 indicates the best possible health. Item #3 is coded in reverse. The items are grouped into 3 domains: Physical (12 items), Emotional (14 items), and Social (13 items). Responses to items #6 and #9 contribute to Physical and Emotional domain scores. Responses to items #17, #20, #21, #24, #25, and #26 contribute to Physical and Social domain scores. Responses to items #2, #13, #15, and #23 contribute to Emotional and Social domain scores. Item #12 is included in all three domain scores. The scoring of domains, as well as the calculation of the global score, is based on an algorithm provided by the authors of the scale [13]. Higher scores indicate a better quality of life.

The general health-related quality of life questionnaire, SF-36, is made up of 36 items questions. Thirty-five of them are grouped into 8 domains: (1) physical functioning, (2) role physical, (3) bodily pain, (4) general health, (5) vitality, (6) social functioning, (7) role emotional and (8) mental health domains. The mean value of the first four domains makes the Physical Composite Score, and the mean value of the latter four domains makes the Mental Composite Score. Scoring of items is done according to the set algorithm, with the scores ranging from 0 (worst possible score) to 100 (best possible score). This questionnaire has been translated and validated in Serbian language. The SF-36 has been translated and validated in Serbian language [14]. The Cronbach’s alpha coefficients for domains ranged from 0.703 to 0.930.

Depression, Anxiety and Stress Scale-21 is composed of 21 items, of which 7 items are focused on depression symptoms, 7 items explore anxiety symptoms, and 7 items examine the symptoms of stress over the past week. Responses are graded on a scale from 0 (not at all) to 3 (mostly or almost always) and multiplied by two. Higher scores indicate more intense symptoms of depression, anxiety and stress. The DASS-21 has been translated and validated in Serbian language [15]. The Cronbach’s alpha coefficients were 0.892 for the depression domain, 0.863 for the anxiety domain and 0.901 for the stress domain.

### 2.4. Follow-Up

The testing was performed 4 times: in hospital one day before CABG (baseline), one month after CABG, 6 months after CABG and 12 months after CABG. The first check-up after discharge was scheduled after 30 days (second time point for questionnaire administration, the first testing after CABG) with the cardiac surgeon. The following health checks were scheduled with a cardiologist in the patients’ primary or secondary health center corresponding to their region of residence. At 6 and 12 months after CABG, patients were contacted via telephone by the same cardiac surgeon who led the recruitment process and tested them for the third and fourth time.

### 2.5. Data Analysis

The description of the study sample was performed using percentages for categorical variables and medians with corresponding interquartile ranges for continuous variables, as the Kolmogorov–Smirnov test suggested a deviation from the normal distribution. The MacNew HRQL scores were presented as means, because they were normally distributed.

Differences in continuous variables were tested using the Mann–Whitney test. Differences in categorical variables were tested using the chi-square test, chi-square linear-by-linear association (for variables with more than two categories) and Fisher’s exact test (for variables with <5 observations per cell).

The internal consistency of the MacNew HRQL in Serbian was tested using the Cronbach’s alpha coefficient (>0.70 adequate) and McDonald’s omega coefficient (>0.70 adequate) [16,17].

Because of several measurements across time, we analyzed sensitivity to change by calculating the standardized effect sized between measurements post-CABG and baseline (pre-CABG) [18]. These were calculated by successively subtracting baseline score from each post-CABG score and dividing it by the standard deviation of the corresponding post-CABG score. Effect size is interpreted as >0.20—small change, >0.50—moderate and >0.80—large change [19].

Additionally, standardized response mean was calculated as mean change/standard deviation of change score. Values are interpreted in the same manner as the values for the effect sizes. We hypothesized that the sensitivity to change would be large from baseline to all three post-CABG measurements.

To check the structural validity, the confirmatory factor analysis (CFA) was applied. We tested several estimators to have a better overview of fit. The following estimators were tested: Maximum Likelihood (ML) and robust ML, Unweighted Least Squares (ULS), Weighted Least Squares (WLS) and diagonally weighted least squares (DWLS). The models were unable to converge when robust ML, DWLS and WLS were tested. We analyzed indices with both ML and ULS estimator.

The optimum indices on CFA that indicate good model fit are: Goodness of fit index (GFI) > 0.95, Comparative fit index (CFI) > 0.95, Tucker–Lewis index (TLI) > 0.95, RMSEA < 0.05 and standardized root mean square residual (SRMR) < 0.05. Acceptable values of GFI, CFI and TLI are >0.90 and those of RMSEA and SRMR are < 0.08 [20].

Longitudinal measurement invariance was also analyzed. We tested a series of models with constrained parameters—configural, metric, scalar, and strict and structural models. Changes in indices across these models (Δ χ^2^, Δ CFI, Δ TLI, Δ RMSEA) were recorded. Thresholds were |ΔCFI| ≤ 0.010, |ΔTLI| ≤ 0.010, and |ΔRMSEA| ≤ 0.015 [21].

To verify true number of factors within the MacNew HRQL in Sebian, we ran Horn’s parallel analysis to remedy potential sampling bias [22]. The syntax [23] to perform the analysis in SPSS (v 31.0.2.0) was available online. The Exploratory Factor Analysis (EFA) with oblique Promax rotation was performed (common extraction method: Maximum Likelihood) to examine the distribution of items when a 2-factor structure was specified based on the parallel analysis. Factor loadings from the pattern matrix were analyzed.

The MacNew global score was correlated with age, sex (coded as 1-male, 2-female), BMI, NYHA class, CCS angina grading scale and LVEF (Spearman’s correlation), as well as with PCS and MCS from the SF-36 and depression, anxiety and stress from the DASS-21. In this way, construct validity was tested. We hypothesized that MacNew global score would positively correlate (Spearman’s rho 0.20–0.40) with NYHA, CCS, PCS and MCS. We hypothesized that MacNew global score would negatively correlate (Spearman’s rho 0.20–0.40) with depression, anxiety and stress as measured by the DASS-21.

Analyses were performed in the Statistical Package for Social Sciences (SPSS Inc., Chicago, IL, USA), version 27. The CFA was conducted in the JASP, version 0.14.0.0 (http://jasp-stats.org, JASP Team (2021). JASP (Version 0.14.8) [Computer software], Amsterdam, The Netherlands) using structural equation modeling (SEM). A probability level of 0.05 was the margin of statistical significance.

## 3. Results

### 3.1. Study Population

**Figure 1:**
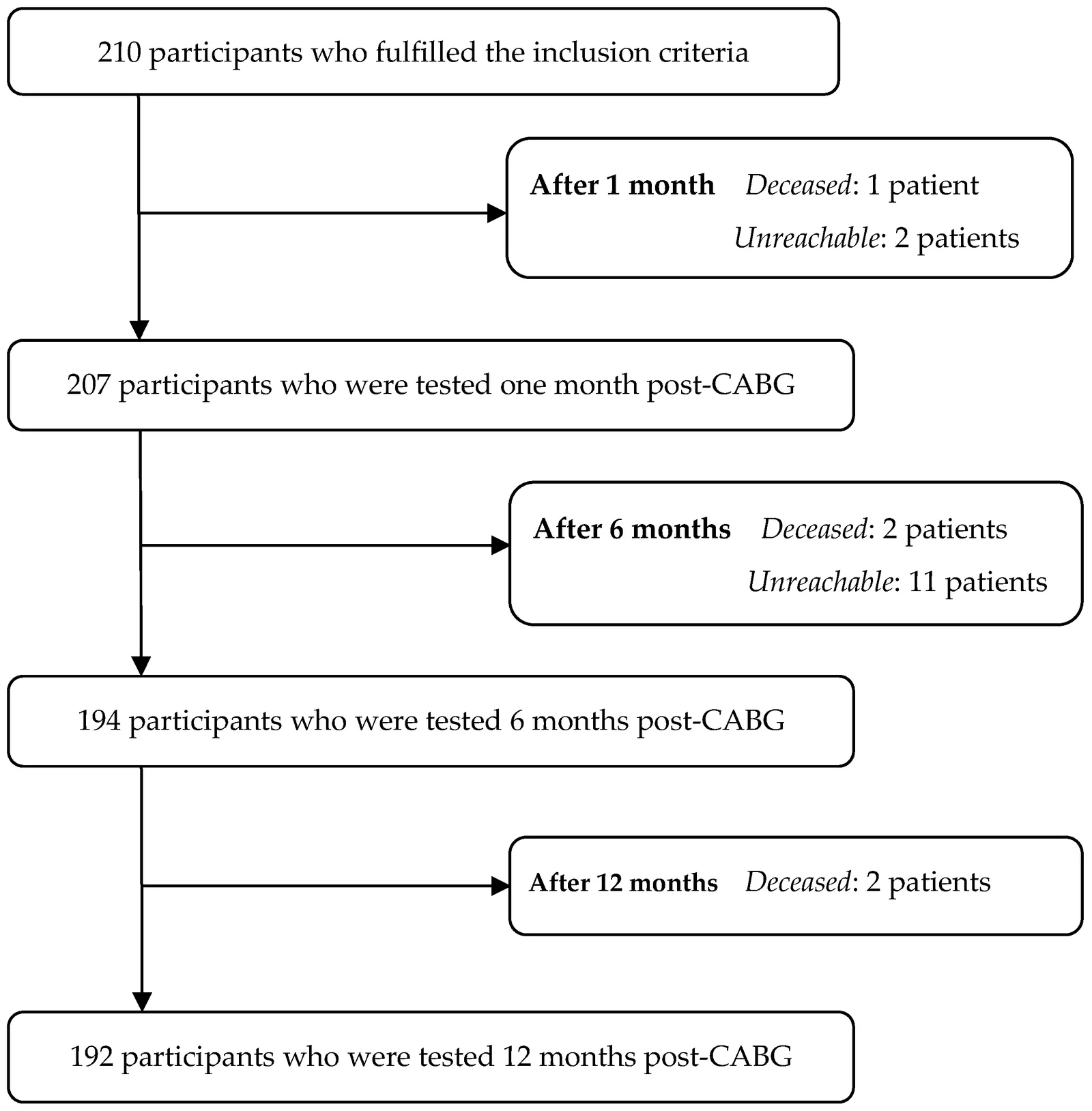
Follow-up of study participants.

A total of 192 patients whose data were collected at all four time points were included in this analysis (Figure 1). Patients who could not be reached at months 1 and 6 through any point of contact, including the telephone number that they reported upon hospital admission, were permanently lost to follow-up.

Demographic and clinical characteristics of the study cohort are shown in Table 1. Most patients were male and, on average, 64.1 ± 7.6 years old. The majority of patients were overweight and obese and were classified per NYHA as class 2 or 3. In terms of the CCS grading, about one-half of the patients had grade 1 and 2 angina. Two-thirds of the study cohort had LVEF > 40%. The majority of patients in this study had hypertension and hyperlipidemia. About one-half had a previous myocardial infarction, while about one-third smoked tobacco and had confirmed type 2 diabetes (Table 1).

The patients had from 1 to 4 diseased coronary vessels. However, most patients had three diseased coronary vessels (71.9%) and most of them received three grafts. The grafts were by and large saphenous vein graft and left internal mammary artery.

Increasing scores across time were observed for general HRQL, i.e., physical and mental quality of life. Decreasing scores over time were observed for depression, anxiety and stress (Table 2).

Over 12 months of follow-up, five patients (2.6%) died (one due to cardiac causes and four due to non-cardiac causes), two patients (1.0%) had minor cerebrovascular insult (CVI), two patients (1.0%) needed percutaneous coronary intervention (PCI), and two patients (1.0%) needed re-hospitalization because of pleural effusion and cardiac decompensation.

Comparison of baseline demographic and clinical characteristics between the patients who completed the follow-up and those who were lost to follow-up is presented in Table 3. The patients did not differ except for the family history of myocardial infarction.

### 3.2. MacNew HRQL Scores and Sensitivity to Change

Table 4 displays the scores on the Serbian MacNew HRQL according to domains. The lowest global and domain scores were observed at baseline, just before the CABG. The highest global and domain scores were observed 12 months after CABG. The lowest domain score on baseline was in the social domain. One month after CABG, the lowest score was seen in the physical domain. Six and 12 months after CABG, the lowest scores were reported in the emotional domain (Table 4). Mean scores, standard deviations and skewness values according to each item at all four measurement time points are presented in Table S1.

It can be observed that the ceiling effect was pronounced for the physical and social domains across time. Floor and ceiling effects according to items are presented in Table S2. At baseline, the largest floor effect was observed for #22 item (Overprotective family). At 12 months post-CABG, the largest floor effect was again observed for item #22 and for item #17 (Sports/exercise limited). With regard to the ceiling effect, the largest effect on baseline was observed for item #19 (Dizzy/lightheaded). The largest ceiling effect at 12 months post-CABG was recorded for item #25 (Unable to socialize).

With regard to the effect size, it can be seen in Table 4 that all changes after CABG had very large effect sizes compared to baseline score.

### 3.3. Internal Consistency Coefficients

Table 5 shows α and ω coefficients for the MacNew HRQL. The coefficients for domains and global score were good (>0.70) across four time points, suggesting excellent internal consistency of the questionnaire. Analysis if-item-deleted suggested that α and ω coefficients remain consistent, while oscillation of values occurs at the second or third decimal place (Table S3). According to the corrected item-total correlation analysis, the lowest coefficient of 0.234 was found for item #3 (Feeling confident) on baseline. The highest coefficient of 0.852 was found for item #26 (Physical activity) 12 months after CABG (Table S3).

### 3.4. Structure Validity

Indices on the CFA based on the original three-factor model with the ML estimator were not acceptable. However, when the ULS estimator was applied the indices at 6 and 12 months post-CABG were good (Table 6). To better understand the distribution of items of the MacNew HRQL in the study cohort the Horn’s parallel analysis was performed.

It was determined that the true number of factors on all four measurements was two (Table S4). Following this, the EFA with two factors was run (Kaiser-Meier-Olkin measure of sampling adequacy was good—0.919 for baseline measurement, 0.921 at 1 month, 0.925 at 6 months and 0.929 at 12 months post-CABG. Bartlett’s test of sphericity level of probability was *p* < 0.001 for all four measurements suggesting that conditions to run the EFA were met) (Table S5).

It was observed that one factor represented the Emotional domain, while the other factor was represented as a combination of the Social and the Physical domains. However, there were slight deviations from this distribution as well. We run the CFA with a two-factor structure where items were distributed to two domains according to the EFA as presented in Table S5. Again, the indices on the CFA were inadequate using the ML estimator. However, using the ULS suggested that indices at 12 months post-CABG were acceptable (Table 6).

### 3.5. Longitudinal Measurement Invariance

Longitudinal measurement invariance was tested using the ML estimator because when the ULS estimator was used the model was unable to converge (Table 7). The indices from the CFA suggested that models were not supported, because the changes in the indices from one model to the next (starting with configural-metric model difference until strict-structural model difference) were larger than the pre-specified values expected in a good model fit. This means that the underlying construct of the MacNew HRQL across time was not consistent.

### 3.6. Construct Validity

Spearman’s correlation of the MacNew HRQL global score with demographic and clinical characteristics is presented in Table 8. A higher MacNew HRQL global score correlated with being male at 1, 6 and 12 months after CABG. A higher MacNew HRQL global score correlated with lower NYHA class and lower angina grade across all four time points. The MacNew HRQL global score did not correlate with LVEF or BMI (Table 8).

Spearman’s correlation of the MacNew HRQL global score with general HRQL questionnaire SF-36 are shown in Table 9. Spearman’s correlation coefficient rho suggested that a higher MacNew HRQL global score correlated with a higher PCS and MCS over time (Table 9).

Spearman’s correlation of the MacNew HRQL global score with general HRQL questionnaire SF-36 are shown in Table 10. A higher MacNew HRQL global score correlated with lower depression, anxiety and stress scores over time (Table 10).

### 3.7. Correlations of Scores at Different Time Points

The correlations of domain scores and total scores are shown in Table 11. It can be seen that correlations of domains and global scores remain stable over time, as scores after 1, 6 and 12 months were correlated with the baseline domain scores, except the Physical domain score on baseline and Physical domain score after 12 months. The global score on baseline correlated with the three subsequent scores (Table 11).

## 4. Discussion

This study adds data to the body of literature on the psychometric properties of the MacNew HRQL [7,8,11,24,25,26,27,28,29,30]. Multiple measurements enabled us to observe the increases in emotional, social and physical domain scores from pre-CABG testing throughout the first year of recovery following CABG. Despite the evidence, as per longitudinal measurement invariance, that questionnaire structure was not the same across time. The three-factor structure after 6 and 12 months seems to mirror the structure of the original MacNew HRQL. Almost all domain scores (except the Physical domain score at baseline and 12 months after CABG) and the global scores remained correlated across time. High internal consistency coefficients for the MacNew HRQL domains and global score were evident as well as good construct validity.

The values of alpha and omega coefficients across all four measurements suggested that the patients in our cohort responded consistently to items pertaining to physical, emotional and social well-being. Over time, the item and domain scores increased with time elapsed since CABG. The increase in domain scores showed a large effect size from the baseline to the end of follow-up, which was expected. The highest floor effect on baseline pertained to overprotective family, suggesting that many patients in the cohort had family support throughout the surgical treatment and could probably rely on their assistance with recovery and daily functioning. This floor effect remained the largest until the end of follow-up. At month 12 after CABG additional large floor effect was recorded for sports and exercise. This finding suggests that some of the patients in the cohort did not engage in intense physical activity. This may depend on the level of physical activity before CABG, fear of physical activity as a trigger for CAD-related complications, or older age. To have a more detailed insight into family support and physical exercise throughout recovery from CABG, it would be helpful to collect qualitative data from both patients and family caregivers as well.

The largest ceiling effect before CABG was observed for dizziness or lightheadedness. This ceiling effect remained the largest at 1 month and 6 months after CABG, suggesting that many patients did not experience those physical sensations associated with CAD. At month 12 after CABG being able to socialize had the largest ceiling effect, highlighting the relevance of personal social network and active contact with family and friends one year after a major open-heart surgery. The role of social contacts and activities one year after surgery seems to be of great importance for our patients and should be further explored in a qualitative and quantitative manner.

When performing psychometric evaluation, most other authors conducted the EFA, but did not perform the CFA, to confirm structure validity of the MacNew HRQL [11,24,25,27,28,29,30]. When the CFA was performed, psychometric evaluations in German and Irish populations showed that the MacNew HRQL could not fit a three-factor model, but identified four- or five-factor structures [8,30]. In this study, after the parallel analysis, it was found that the responses of patients in our cohort to the MacNew HRQL have two-factor structure across all four measurements. The physical domain was identified first; however, it seemed that the emotional and social domains merged into a single domain. In contrast, on the CFA the two-factor model did not have acceptable indices and had to be rejected. Nevertheless, the indices of the three-factor model using the ULS had good characteristics when the MacNew HRQL was administered 6 and 12 months after CABG, probably suggesting that after early recovery from open heart surgery, our patients returned to their habitual daily activities and could therefore distinguish emotional from social functioning on the one hand and physical functioning on the other.

Neither the three-factor nor two-factor structure was supported by the CFA at baseline or 1 month after CABG. Study participants went through a surgical procedure which involved median sternotomy and extracorporeal circulation, requiring at least three months for full recovery after median sternotomy [31] and they may have experienced intense postoperative pain around the surgical wound. This result may partially explain why the original three-factor structure (emotional, social, physical domains) was not supported by the model 1 month after CABG. Further, patients’ internal standards and conceptualization of HRQL could have changed after such major open-heart surgery.

The construct validity of the MacNew HRQL in Serbian language was good. This means that MacNew HRQL scores mirror the increases and decreases in scores on other well-established and widely used questionnaires such as the general HRQL questionnaire SF-36 and depression/anxiety/stress questionnaire DASS-21. Such evidence is quite helpful because it shows that the specific HRQL questionnaire can adequately record self-reported physical and mental health of patients who underwent elective CABG. As a result, the MacNew HRQL can be confidently used in situations that require comprehensive evaluation of health status of patients with CAD who are preparing for or who have already had elective CABG.

Regarding the study limitations, patients with CAD who participated in this study came from diverse socio-economic backgrounds, because our institution is one of five centers for cardiac surgery in the country which are all state-run and publicly financed. Because people in Serbia have universal health care access, virtually all open heart surgeries are performed in one of these five centers. Nevertheless, because these patients underwent elective CABG and numerous chronic illnesses and conditions were excluded, our results cannot be generalized. Additionally, patients who needed emergency CABG may have had a different perception of HRQL. Furthermore, the study cohort was composed mostly of males (around 20% were females), which can affect the interpretation of the scores and may not represent women’s perceptions. The questionnaires were administered first face-to-face during pre-CABG testing and later patients were tested over telephone. Although one researcher tested the same patients at all four time points, we cannot rule out potential bias due to different modes of questionnaire administration. Minimal clinical difference testing was not included in the present analysis. While efforts were made to organize as many repeated measurements as possible, issues with graft patency and recurrence of angina over longer intervals (at least 5 years) could affect the results. Long-term follow-up is the preferred modality for analyzing outcomes of various surgical interventions.

## 5. Conclusions

In conclusion, the MacNew HRQL in Serbian language has good internal consistency coefficients and construct validity across all four time points. The three-factor structure with emotional, social and physical domains was most favorable under the ULS at 6 and 12 months post-CABG. However, the factor structure before and at 1-month post-CABG might be problematic. The longitudinal model showed inadequate fit and measurement invariance was not supported. Therefore changes in scores over time cannot be interpreted unequivocally as changes in the same underlying constructs. The scores just before and after CABG should be interpreted with caution. Bearing in mind its good internal consistency and construct validity, the MacNew HRQL could be used to evaluate HRQL in patients before and after CABG.

## Figures and Tables

**Table 1:** Socio-demographic and clinical characteristics of the study cohort on baseline (N = 192).

Variables	Count	Percentage
Age	64.1 ± 7.6 *	
Sex		
Male	154	80.2
Female	38	19.8
Body mass index category		
Normal	40	20.8
Overweight	100	52.1
Obese	52	27.1
New York Heart Association class		
1	29	15.1
2	97	50.5
3	66	34.4
Canadian Cardiovascular Society angina grade		
1	12	6.2
2	89	46.4
3	47	24.5
4	44	22.9
Left ventricle ejection fraction		
≤40	70	36.8
41–49	33	17.4
≥50	87	45.8
Diabetes mellitus	73	38.0
Hypertension	179	93.2
Hyperlipedemia	175	91.1
Previous myocardial infarction	86	44.8
No. of diseased coronary vessels		
1	2	1.0
2	27	14.1
3	138	71.9
4	25	13.0
Potentially non-graftable arteries	23	12.0
No. of grafts		
1	3	1.6
2	37	19.3
3	122	63.5
4–5	30	15.6
Type of graft		
SVG	184	95.8
LIMA	188	97.9
RIMA	7	3.6
Radial graft artery	18	9.4
Family history of cardiovascular disease	130	67.7
Smoking	60	31.2

Legend: * refers to mean ± standard deviation; SVG—saphenous vein graft; LIMA—left internal mammary artery; RIMA—right internal mammary artery; CVD—cardiovascular diseases.

**Table 2:** Scores on the Short Form-36 and the Depression Anxiety Stress Scale-21 on baseline (N = 192).

Variables	Median (Interquartile Range)
Physical Composite Score on baseline	43.1 (20.9)
Physical Composite Score after 1 month	71.6 (36.9)
Physical Composite Score after 6 months	87.2 (21.2)
Physical Composite Score after 12 months	90.0 (15.9)
Mental Composite Score on baseline	53.4 (31.8)
Mental Composite Score after 1 month	84.2 (30.3)
Mental Composite Score after 6 months	92 (15.7)
Mental Composite Score after 12 months	94.0 (14.0)
Depression score on baseline	8.0 (10.0)
Depression score after 1 month	0.0 (4.0)
Depression score after 6 months	0.0 (2.0)
Depression score after 12 months	0.0 (0.0)
Anxiety score on baseline	8.0 (10.0)
Anxiety score after 1 month	2.0 (5.5)
Anxiety score after 6 months	0.0 (4.0)
Anxiety score after 12 months	0.0 (4.0)
Stress score on baseline	14.0 (10.0)
Stress score after 1 month	4.0 (12.0)
Stress score after 6 months	2.0 (6.0)
Stress score after 12 months	0.0 (6.0)

**Table 3:** Comparison of baseline demographic and clinical characteristics of the study cohort and patients who were lost to follow-up.

Variables	Complete Follow-Up	Lost to Follow-Up	*p* for Difference
	Count (%) N = 192	Count (%) N = 18	
Age *	64.1 ± 7.6	66.0 ± 7.0	0.399
Sex			
Male	154 (80.2)	15 (83.3)	0.518
Female	38 (19.8)	3 (16.7)	
Body mass index category			
Normal	40 (20.8)	3 (16.7)	0.351
Overweight	100 (52.1)	8 (44.4)	
Obese	52 (27.1)	7 (38.9)	
New York Heart Association class			
1	29 (15.1)	2 (11.1)	0.610
2	97 (50.5)	9 (50.0)	
3	66 (34.4)	7 (38.9)	
Canadian Cardiovascular Society angina grade			
1	12 (6.2)	0 (0)	0.712
2	89 (46.4)	9 (50.0)	
3	47 (24.5)	5 (27.8)	
4	44 (22.9)	4 (22.2)	
Left ventricle ejection fraction			
≤40	70 (36.8)	7 (38.9)	0.657
41–49	33 (17.4)	4 (22.2)	
≥50	87 (45.8)	7 (38.9)	
Diabetes mellitus	73 (38.0)	10 (55.60)	0.159
Hypertension	179 (93.2)	18 (100.0)	0.609
Hyperlipedemia	175 (91.1)	18 (100.0)	0.372
Previous myocardial infarction	86 (44.8)	10 (55.60)	0.461
No. of diseased coronary vessels			
1	2 (1.0)	0 (0)	0.534
2	27 (14.1)	3 (16.7)	
3	138 (71.9)	11 (61.1)	
4	25 (13.0)	4 (22.2)	
Potentially non-graftable arteries	23 (12.0)	0 (0)	0.453
No. of grafts			
1	3 (1.6)	0 (0)	0.462
2	37 (19.3)	3 (16.7)	
3	122 (63.5)	11 (61.1)	
4–5	30 (15.6)	4 (22.2)	
Type of graft			
Saphenous vein graft	184 (95.8)	18 (100.0)	0.462
Left internal mammary artery	188 (97.9)	18 (100.0)	0.697
Right internal mammary artery	7 (3.6)	0 (0)	0.529
Radial artery	18 (9.4)	2 (11.1)	0.683
Family history of cardiovascular disease	130 (67.7)	8 (44.4)	0.047
Smoking	60 (31.2)	4 (22.2)	0.978

Legend: * refers to mean ± standard deviation.

**Table 4:** Scores, floor and ceiling effects and effect size of the original 26-item MacNew Heart Disease Health-Related Quality of Life Questionnaire in Serbian language across time points (min–max range 1–7).

MacNew Domains	Baseline (Pre-CABG)	1 Month After CABG			6 Months After CABG			12 Months After CABG		
	Mean ± SD	Mean ± SD	ES	SRM	Mean ± SD	ES	SRM	Mean ± SD	ES	SRM
Physical domain	4.43 ± 1.37	5.41 ± 1.23	0.79	0.58	6.13 ± 1.03	1.65	1.03	6.34 ± 0.95	2.01	1.19
Floor effect (%)	0.5	0.5			1.0			1.0		
Ceiling effect (%)	1.0	4.7			14.6			22.9		
Emotional domain	4.62 ± 1.21	5.64 ± 0.89	1.14	0.72	5.99 ± 0.73	1.87	1.00	6.10 ± 0.65	2.76	1.12
Floor effect (%)	0.5	1.0			0.5			0.5		
Ceiling effect (%)	1.6	0.5			0.5			1.0		
Social domain	4.40 ± 1.45	5.45 ± 1.28	0.82	0.58	6.15 ± 1.02	1.71	1.05	6.35 ± 0.94	2.07	1.19
Floor effect (%)	0.5	0.5			0.5			0.5		
Ceiling effect (%)	2.1	8.3			17.2			24.5		
Global score	4.50 ± 1.21	5.49 ± 1.01	0.90	0.62	6.03 ± 0.83	1.84	1.09	6.19 ± 0.76	2.22	1.24
Floor effect (%)	1.0	0.5			0.5			0.5		
Ceiling effect (%)	0.5	3.1			0.5			0.1		

Legend: CABG—coronary artery bypass grafting; SD—standard deviation; ES—effect size expressed by Cohen’s d, values are calculated relative to baseline score; SRM—standardized response mean.

**Table 5:** Internal consistency coefficients of the original 26-item MacNew Heart Disease Health-Related Quality of Life Questionnaire (MacNew HRQL) in Serbian language across time.

MacNew HRQL Domains		Baseline (Pre-CABG)	1 Month After CABG	6 Months After CABG	12 Months After CABG
Physical domain	ω (95%CI)	0.929 (0.922–0.936)	0.925 (0.919–0.932)	0.937 (0.926–0.943)	Table
	α (95%CI)	0.926 (0.920–0.931)	0.915 (0.909–0.922)	0.933 (0.925–0.941)	0.950 (0.942–0.957)
Emotional domain	ω (95%CI)	0.937 (0.932–0.945)	0.934 (0.927–0.942)	0.941 (0.935–0.947)	0.934 (0.926–0.943)
	α (95%CI)	0.919 (0.913–0.925)	0.892 (0.885–0.900)	0.933 (0.924–0.942)	0.944 (0.937–0.951)
Social domain	ω (95%CI)	0.933 (0.926–0.940)	0.936 (0.930–0.941)	0.933 (0.925–0.941)	0.935 (0.928–0.941)
	α (95%CI)	0.931 (0.926–0.937)	0.899 (0.892–0.907)	0.930 (0.925–0.937)	0.946 (0.939–0.951)
Global score	ω (95%CI)	0.955 (0.948–0.965)	0.955 (0.942–0.966)	0.958 (0.951–0.964)	0.958 (0.952–0.964)
	α (95%CI)	0.935 (0.928–0.945)	0.891 (0.884–0.899)	0.932 (0.924–0.940)	0.947 (0.932–0.955)

Legend: α—Cronbach’s alpha coefficient, ω—McDonald’s omega coefficient. CI—confidence interval.

**Table 6:** Confirmatory factor analysis indices based on the original 3-factor model and 2-factor model suggested by parallel analysis.

Model	χ^2^	df	CFI	TLI	RMSEA (90% CI)	SRMR
Baseline (pre-CABG)						
2-factor (ML)	701.62	229	0.855	0.840	0.104 (0.095–0.112)	0.072
2-factor (ULS)	2191.2	229	0.985	0.984	0.212 (0.204–0.220)	0.061
3-factor (ML)	677.95	283	0.888	0.871	0.085 (0.077–0.094)	0.058
3-factor (ULS)	2176.0	283	0.988	0.956	0.187 (0.183–0.195)	0.055
1 month after CABG						
2-factor (ML)	1170.05	298	0.762	0.740	0.123 (0.116–0.131)	0.087
2-factor (ULS)	2564.3	298	0.974	0.972	0.200 (0.192–0.207)	0.083
3-factor (ML)	846.86	283	0.846	0.823	0.102 (0.094–0.110)	0.071
3-factor (ULS)	1453.4	283	0.987	0.985	0.147 (0.140–0.155)	0.066
6 months after CABG						
2-factor (ML)	914.40	298	0.846	0.832	0.104 (0.096–0.111)	0.068
2-factor (ULS)	798.2	298	0.988	0.986	0.094 (0.086–0.102)	0.068
3-factor (ML)	660.34	283	0.906	0.892	0.083 (0.075–0.094)	0.051
3-factor (ULS)	420.2	283	0.997	0.996	0.050 (0.040–0.060)	0.051
12 months after CABG						
2-factor (ML)	1096.93	298	0.809	0.792	0.118 (0.111–0.126)	0.071
2-factor (ULS)	523.3	298	0.992	0.991	0.063 (0.054–0.072)	0.068
3-factor (ML)	845.31	283	0.865	0.845	0.102 (0.094–0.110)	0.056
3-factor (ULS)	304.5	283	0.999	0.999	0.020 (0.000–0.035)	0.056

Legend: CABG—coronary artery bypass grafting; χ^2^—chi-square; df—degrees of freedom; GFI—Goodness of fit index; CFI—Comparative fit index; RMSEA—Root Mean Square Error of Approximation; SRMR—Standardized Root Mean Square Residual. ML—Maximum Likelihood estimator; ULS—Unweighted Least Squares estimator.

**Table 7:** Indices of longitudinal measurement invariance of the original 3-factor model across 4 points of measurement.

Parameters	Models				
	Configural	Metric	Scalar	Strict	Structural
χ^2^ (df)	3030.4 (1132)	3432.3 (1240)	3776.6 (1309)	6030.7 (1387)	6610.3 (1414)
*p* for χ^2^ (df)	0.001	0.001	0.001	0.001	0.001
Δ χ^2^ (df)		401.9 (108)	344.3 (69)	2254.1 (78)	579.6 (25)
*p* for Δ χ^2^ (df)		0.001	0.001	0.001	0.001
CFI	0.876	0.857	0.839	0.698	0.662
ΔCFI		−0.019	−0.018	−0.141	−0.036
TLI	0.858	0.850	0.841	0.717	0.689
ΔTLI		−0.008	−0.009	−0.124	−0.128
RMSEA (90%CI)	0.093 (0.089–0.097)	0.096 (0.092–0.100)	0.099 (0.095–0.103)	0.132 (0.129–0.135)	0.138 (0.135–0.142)
ΔRMSEA		0.003	0.003	0.033	0.006

Legend: χ^2^—chi-square; df—degrees of freedom; GFI—Goodness of fit index; CFI—Comparative fit index; RMSEA—Root Mean Square Error of Approximation; SRMR—Standardized Root Mean Square Residual. Δ change in χ^2^ (df), TLI, CFI and RMSEA.

**Table 8:** Spearman’s correlation of the MacNew Heart Disease Health-Related Quality of Life Questionnaire (MacNew HRQL) global score with demographic and clinical parameters.

Variable		MacNew Global Score			
		Baseline (Pre-CABG)	1 Month After CABG	6 Months After CABG	12 Months After CABG
Sex	rho	−0.128	−0.223	−0.212	−0231
	*p*	0.078	0.002	0.003	0.001
Age	rho	−0.166	0.091	0.035	0.012
	*p*	0.022	0.209	0.630	0.872
BMI	rho	−0.107	0.020	0.100	0.088
	*p*	0.140	0.782	0.168	0.226
NYHA	rho	−0.321	−0.369	−0.347	−0.345
	*p*	0.001	0.001	0.001	0.001
CCS	rho	−0.270	−0.212	−0233	−0.221
	*p*	0.001	0.003	0.001	0.002
LVEF	rho	0.084	0.062	0.054	0.028
	*p*	0.248	0.395	0.458	0.699

Legend: CABG—coronary artery bypass grafting; BMI—body mass index; NYHA—New York Heart Association class; CCS—Canadian Cardiovascular Society angina grade; LVEF—left ventricle ejection fraction.

**Table 9:** Spearman’s correlation of the MacNew Heart Disease Health-Related Quality of Life Questionnaire (MacNew HRQL) and composite scores of the SF-36 across time.

Scales		MacNew Global Score			
		Baseline (Pre-CABG)	1 Month After CABG	6 Months After CABG	12 Months After CABG
PCS baseline	rho	0.619			
	*p*	0.001			
MCS baseline	rho	0.667			
	*p*	0.001			
PCS 1 month after CABG	rho		0.746		
	*p*		0.001		
MCS 1 month after CABG	rho		0.720		
	*p*		0.001		
PCS 6 months after CABG	rho			0.724	
	*p*			0.001	
MCS 6 months after CABG	rho			0.698	
	*p*			0.001	
PCS 12 months after CABG	rho				0.707
	*p*				0.001
MCS 12 months after CABG	rho				0.658
	*p*				0.001

Legend: CABG—coronary artery bypass grafting; SF-36—Short Form 36; PCS—Physical Composite Score of the SF-36; MCS—Mental Composite Score of the SF-36.

**Table 10:** Spearman’s correlation of the MacNew Heart Disease Health-Related Quality of Life Questionnaire (MacNew HRQL) with depression, anxiety and stress scores across time.

Scales		MacNew Global Score			
		Baseline (Pre-CABG)	1 Month After CABG	6 Months After CABG	12 Months After CABG
DASS-D baseline	rho	−0.630			
	*p*	0.001			
DASS-A baseline	rho	−0.522			
	*p*	0.001			
DASS-S baseline	rho	−0.492			
	*p*	0.001			
DASS-D 1 month after CABG	rho		−0.630		
	*p*		0.001		
DASS-A 1 month after CABG	rho		−0.569		
	*p*		0.001		
DASS-S 1 month after CABG	rho		−0.680		
	*p*		0.001		
DASS-D 6 months after CABG	rho			−0.663	
	*p*			0.001	
DASS-A 6 months after CABG	rho			−0.546	
	*p*			0.001	
DASS-S 6 months after CABG	rho			−0.572	
	*p*			0.001	
DASS-D 12 months after CABG	rho				−0.588
	*p*				0.001
DASS-A 12 months after CABG	rho				−0.482
	*p*				0.001
DASS-S 12 months after CABG	rho				−0.509
	*p*				0.001

Legend: CABG—coronary artery bypass grafting; DASS-21—Depression Anxiety Stress Scale-21; DASS-D—depression score of the DASS-21; DASS-A—anxiety score of the DASS-21; DASS-S—stress score of the DASS-21.

**Table 11:** Spearman’s correlation of the MacNew Heart Disease Health-Related Quality of Life Questionnaire (MacNew HRQL) scores across time.

Scores		Baseline MacNew HRQL Scores			
		Physical Domain	Emotional Domain	Social Domain	Global Score
1 month after CABG					
Physical domain	rho	0.182			
	*p*	0.011			
Emotional domain	rho		0.203		
	*p*		0.005		
Social domain	rho			0.191	
	*p*			0.008	
Global score	rho				0.186
	*p*				0.010
6 months after CABG					
Physical domain	rho	0.165			
	*p*	0.023			
Emotional domain	rho		0.164		
	*p*		0.023		
Social domain	rho			0.247	
	*p*			0.001	
Global score	rho				0.198
	*p*				0.006
12 months after CABG					
Physical domain	rho	0.134			
	*p*	0.065			
Emotional domain	rho		0.192		
	*p*		0.008		
Social domain	rho			0.233	
	*p*			0.001	
Global score	rho				0.198
	*p*				0.006

Legend: CABG—coronary artery bypass grafting.

## Data Availability

The dataset underlying this study is available upon request to the corresponding author.

## Supplementary Materials

The following supporting information can be downloaded at: https://www.mdpi.com/article/10.3390/epidemiologia7050144/s1, Table S1. Mean, standard deviation and skewness according to items and domains across time; Table S2. Proportions of minimum (floor effect) and maximum (ceiling effect) scores per item across time; Table S3. Internal consistency coefficients if-item-deleted and corrected item-total correlation (CI-TC) of the original 26-item MacNew Heart Disease Health-Related Quality of Life Questionnaire in Serbian language across time points; Table S4. Comparison of eigenvalues from the Exploratory Factor Analysis (EFA) and parallel analysis; Table S5. Exploratory Factor Analysis with Promax rotation of the MacNew Heart Disease Health-Related Quality of Life Questionnaire (MacNew HRQL) in Serbian language at four time points after parallel analysis with 2 set factors.

## Abbreviations

The following abbreviations are used in this manuscript:

CABG	Coronary artery bypass grafting
CAD	Coronary Artery Disease
CCS	Canadian Cardiovascular Society
CFA	Confirmatory Factor Analysis
DASS 21	Depression Anxiety Stress Scale 21
EFA	Exploratory Factor Analysis
MacNew HRQoL	MacNew Heart Disease Health-Related Quality of Life Questionnaire
NYHA	New York Heart Association
SF-36	Short Form 36
