# Supplementary material for: Psychometric Evaluation of the Serbian MacNew Heart Disease Health-Related Quality of Life Questionnaire Before and After Elective Coronary Artery Bypass Grafting
Source: Epidemiologia (Basel). 2026 Oct 15;7(5):144. doi: 10.3390/epidemiologia7050144 (PMC13703691; doi:10.3390/epidemiologia7050144)
Supplement: Supplementary file 1 [file epidemiologia-07-00144-s001.zip › epidemiologia-4452113-supplementary.pdf]

Table S1. Mean, standard deviation and skewness according to items and domains across time

Items	Baseline (pre-CABG)			1 month after CABG			6 months after CABG			12 months after CABG		
	Mean	SD	Skew	Mean	SD	Skew	Mean	SD	Skew	Mean	SD	Skew
1.Frustrated	4.6	1.6	-0.55	6.0	1.2	-1.64	6.3	0.9	-1.66	6.5	0.8	-2.08
2.Worthless	4.7	1.9	-0.43	6.2	1.2	-2.08	6.5	0.9	-2.66	6.6	0.7	-3.90
3.Confident	4.8	1.8	0.43	5.9	1.3	1.3	6.2	1.1	1.95	6.3	1.0	2.16
4. Down in dumps	4.8	1.7	-0.64	6.0	1.3	-1.71	6.4	0.9	-2.53	6.5	.88	-3.38
5.Relaxed	3.8	1.7	0.19	5.1	1.6	-0.70	5.7	1.3	-1.36	5.8	1.3	-1.52
6. Worn out	4.2	1.6	-0.28	5.3	1.4	-0.77	5.9	1.3	-1.42	6.2	1.1	-1.84
7. Happy with personal life	4.0	1.7	-0.08	5.3	1.2	-0.52	5.6	1.2	-1.03	5.7	1.1	-.83
8. Restless	4.9	1.6	-0.69	5.9	1.2	-1.64	6.4	1.0	-2.29	6.4	0.9	-2.39
9. Shortness of breath	4.7	1.6	-0.19	5.8	1.4	-1.15	6.3	1.1	-1.99	6.4	1.0	-2.49
10. Tearful	5.7	1.6	-1.29	6.4	1.2	-2.68	6.6	0.8	-3.20	6.7	0.7	-3.89
11. More dependent	4.6	1.9	-0.41	5.5	1.7	-1.20	6.2	1.3	-1.91	6.4	1.1	-2.54
12. Social activities	4.1	1.9	-0.15	5.7	1.8	-1.42	6.3	1.2	-2.43	6.5	1.0	-2.91
13. Others/less confidence in you	5.6	1.8	-1.14	6.4	1.1	-2.45	6.5	1.0	-3.31	6.6	1.0	-3.46
14. Chest pain	4.7	1.8	-0.51	6.4	1.0	-2.60	6.6	0.9	-3.67	6.6	0.8	-3.44
15. Lack self-confidence	5.1	1.7	0.74	6.2	1.2	-2.11	6.5	1.0	-2.87	6.6	0.8	-3.27
16. Aching legs	5.1	1.7	-0.85	5.5	1.7	-1.22	5.9	1.6	-1.64	6.1	1.4	-1.98
17. Sports/exercise limited	3.6	2.1	0.29	4.0	2.1	-0.41	5.3	1.8	-1.00	5.6	1.8	-1.35
18. Frightened	4.7	1.7	-0.60	5.9	1.5	-1.60	6.3	1.2	-2.27	6.5	1.0	-2.88
19. Dizzy or lightheaded	6.1	1.3	-1.80	6.4	1.1	-2.46	6.6	0.9	-3.34	6.6	0.8	-3.55
20. Restricted or limited	4.1	1.9	-0.04	5.2	1.6	-0.84	6.1	1.2	-1.77	6.3	1.1	-2.29
21. Unsure about exercise	4.0	2.0	-1.26	4.4	2.0	-0.27	5.5	1.7	-1.23	5.9	1.6	-1.66
22. Overprotective family	2.9	1.9	0.82	4.2	2.2	-0.12	5.2	2.0	-0.76	5.5	1.8	-1.06
23. Burden on others	5.4	1.9	-1.05	6.0	1.5	-1.57	6.4	1.1	-2.75	6.5	1.0	-3.30
24. Excluded	4.9	2.1	-0.59	6.0	1.7	-1.75	6.4	1.3	-2.58	6.6	1.0	-3.31

25. Unable to socialize	5.9	2.0	-0.57	5.9	1.7	1.79	6.4	1.2	-2.69	6.6	1.0	-3.53
26. Physical activity	3.9	1.9	0.04	4.8	1.8	1.81	5.9	1.4	-1.57	6.2	1.2	-2.13
Physical domain	4.5	1.4	-0.23	5.4	1.2	-0.95	6.1	1.0	-1.91	6.3	0.9	-2.47
Emotional domain	4.6	1.2	-0.72	5.6	0.9	-1.61	6.0	.7	-2.30	6.1	0.6	-2.77
Social domain	4.4	1.4	-0.35	5.4	1.2	-0.99	6.1	1.0	-1.81	6.3	0.9	-2.42
Global score	4.5	1.2	-0.46	5.5	1.0	-1.17	6.0	.83	-1.97	6.1	0.7	-2.52

Legend: SD – standard deviation; Skew – skewness.

Table S2. Proportions of minimum (floor effect) and maximum (ceiling effect) scores per item across time

Items	Baseline (pre-CABG)		1 month after CABG		6 months after CABG		12 months after CABG	
	Floor effect (%)	Ceiling effect (%)	Floor effect (%)	Ceiling effect (%)	Floor effect (%)	Ceiling effect (%)	Floor effect (%)	Ceiling effect (%)
1.Frustrated	5.7	10.4	1.0	49.5	0.5	60.4	0.5	67.2
2.Worthless	6.3	22.9	1.6	61.5	0.5	68.8	0.5	78.6
3.Confident	4.2	22.4	3.6	42.7	0.5	54.2	0.5	56.3
4. Down in dumps	4.7	15.6	1.0	52.1	0.5	66.1	0.5	73.4
5.Relaxed	6.8	7.8	1.6	21.9	1.0	36.5	1.0	39.6
6. Worn out	5.7	3.6	1.0	25.5	0.5	49.0	2.6	54.7
7. Happy with personal life	9.4	6.8	2.1	18.8	1.0	28.6	0.5	29.7
8. Restless	3.6	15.1	1.0	43.2	1.6	62.5	1.0	66.1
9. Shortness of breath	2.6	20.8	1.0	47.9	0.5	65.1	0.5	76.0
10. Tearful	2.1	46.9	1.0	74.5	0.5	80.7	0.5	83.3
11. More dependent	9.4	20.3	4.2	43.2	3.6	64.1	2.1	71.9
12. Social activities	12.0	10.9	5.2	52.1	0.5	71.4	0.5	77.1
13. Others/less confidence in you	4.7	49.0	0.5	72.4	1.0	80.2	1.0	81.3
14. Chest pain	6.8	20.8	0.5	71.9	0.5	79.2	1.0	82.8
15. Lack self-confidence	4.2	27.6	1.6	63.0	0.5	74.0	0.5	79.7
16. Aching legs	5.7	25.0	4.7	38.0	2.1	60.4	1.0	66.1
17. Sports/exercise limited	21.9	14.1	18.2	20.8	6.8	38.0	5.2	49.5
18. Frightened	6.3	15.6	1.6	55.2	0.5	69.8	0.5	75.0
19. Dizzy or lightheaded	1.6	54.7	0.5	74.5	0.5	82.3	0.5	81.3
20. Restricted or limited	9.9	10.4	2.6	28.1	0.5	57.3	0.5	68.2
21. Unsure about exercise	14.2	11.5	13.0	21.9	4.7	39.6	4.2	57.3
22. Overprotective family	34.4	7.8	16.2	27.1	5.2	44.3	5.2	46.9
23. Burden on others	6.8	47.9	0.5	62.0	0.5	75.5	0.5	79.2
24. Excluded	7.3	35.9	3.6	69.8	0.5	79.7	0.5	54.4

25. Unable to socialize	7.3	30.7	2.6	67.2	0.5	78.6	0.5	86.5
26. Physical activity	12.5	8.9	5.2	24.0	1.6	54.7	0.5	63.5

Table S3. Internal consistency coefficients if-item-deleted and corrected item-total correlation (CI-TC) of the original 26-item MacNew Heart Disease Health-Related Quality of Life Questionnaire in Serbian language across time points

Items	Baseline (pre-CABG)			1 month after CABG			6 months after CABG			12 months after CABG		
	α if item deleted	ω if item deleted	CI-TC	α if item deleted	ω if item deleted	CI-TC	α if item deleted	ω if item deleted	CI-TC	α if item deleted	ω if item deleted	CI-TC
1.Frustrated	0.948	0.952	0.682	0.943	0.949	0.643	0.945	0.952	0.606	0.946	0.953	0.509
2.Worthless	0.948	0.952	0.716	0.943	0.950	0.629	0.944	0.952	0.673	0.945	0.953	0.650
3.Confident	0.958	0.958	0.234	0.953	0.955	0.499	0.956	0.958	0.583	0.956	0.958	0.559
4. Down in dumps	0.948	0.952	0.735	0.942	0.949	0.658	0.943	0.951	0.766	0.944	0.952	0.756
5.Relaxed	0.949	0.953	0.600	0.943	0.950	0.570	0.944	0.952	0.655	0.944	0.952	0.640
6. Worn out	0.948	0.952	0.720	0.942	0.949	0.704	0.942	0.949	0.806	0.942	0.950	0.824
7. Happy with personal life	0.949	0.952	0.643	0.944	0.950	0.543	0.944	0.952	0.614	0.945	0.953	0.555
8. Restless	0.948	0.952	0.775	0.942	0.949	0.696	0.944	0.952	0.690	0.944	0.952	0.713
9. Shortness of breath	0.948	0.952	0.693	0.943	0.949	0.630	0.944	0.951	0.669	0.944	0.951	0.715
10. Tearful	0.950	0.954	0.493	0.943	0.950	0.639	0.945	0.952	0.563	0.945	0.953	0.564
11. More dependent	0.948	0.952	0.710	0.941	0.947	0.780	0.943	0.950	0.770	0.943	0.951	0.784
12. Social activities	0.947	0.951	0.770	0.940	0.946	0.827	0.942	0.949	0.809	0.943	0.951	0.788
13. Others/less confidence in you	0.948	0.952	0.703	0.943	0.950	0.573	0.945	0.952	0.600	0.944	0.952	0.638
14. Chest pain	0.948	0.952	0.676	0.944	0.950	0.481	0.944	0.951	0.666	0.944	0.952	0.672
15. Lack self-confidence	0.947	0.951	0.818	0.942	0.948	0.768	0.943	0.951	0.766	0.943	0.951	0.786
16. Aching legs	0.949	0.953	0.582	0.944	0.951	0.548	0.945	0.952	0.626	0.945	0.953	0.604
17. Sports/exercise limited	0.949	0.953	0.631	0.942	0.949	0.696	0.944	0.951	0.698	0.944	0.952	0.708
18. Frightened	0.948	0.952	0.748	0.942	0.949	0.673	0.943	0.951	0.721	0.943	0.951	0.740
19. Dizzy or lightheaded	0.950	0.953	0.556	0.943	0.950	0.566	0.945	0.952	0.521	0.945	0.953	0.540
20. Restricted or limited	0.948	0.951	0.733	0.942	0.948	0.692	0.943	0.950	0.759	0.942	0.950	0.810
21. Unsure about exercise	0.948	0.952	0.699	0.941	0.948	0.724	0.944	0.952	0.665	0.944	0.952	0.686

22. Overprotective family	0.951	0.955	0.432	0.945	0.952	0.540	0.948	0.956	0.466	0.949	0.957	0.468
23. Burden on others	0.948	0.952	0.675	0.941	0.948	0.727	0.944	0.951	0.699	0.944	0.952	0.708
24. Excluded	0.948	0.952	0.682	0.942	0.949	0.691	0.943	0.950	0.739	0.943	0.951	0.751
25. Unable to socialize	0.948	0.951	0.737	0.940	0.947	0.796	0.942	0.949	0.819	0.943	0.951	0.817
26. Physical activity	0.948	0.952	0.675	0.941	0.948	0.752	0.942	0.949	0.809	0.942	0.949	0.852

Table S4. Comparison of eigenvalues from the Exploratory Factor Analysis (EFA) and parallel analysis

Measurement	EFA Eigenvalues	Parallel Eigenvalues	True number of factors
Pre-CABG			
	12.733	1.744	2
	2.223	1.633	
	1.224	1.541	
	1.091	1.456	
1 month after CABG			
	12.586	1.727	2
	2.220	1.612	
	1.432	1.539	
	1.057	1.416	
6 months after CABG			
	13.513	1.755	2
	2.264	1.614	
	1.305	1.572	
12 months after CABG			
	13.658	1.704	2
	2.058	1.273	
	1.286	1.596	

Legend: True factor is determined when parallel Eigenvalue is lower that EFA Eigenvalue

Table S5. Exploratory Factor Analysis with Promax rotation of the MacNew Heart Disease Health-related Quality of Life questionnaire (MacNew HRQL) in Serbian language at four time points after parallel analysis with 2 set factors

Items	Baseline (pre-CABG)		1 month after CABG		6 months after CABG		12 months after CABG	
	F1-Emo	F2-Soc/Phys	F1-Emo	F2-Soc/Phys	F1-Emo	F2-Soc/Phys	F1-Emo	F2-Soc/Phys
1.Frustrated	**.811**	-.031	**.690**	.076	**.752**	-.020	**.813**	-.197
2.Worthless	**.790**	.032	**.920**	-.163	**.846**	-.036	**.818**	-.038
3.Confident	**.583**	.311	**.937**	.335	**.714**	.014	**.672**	.010
4. Down in dumps	**.911**	-.071	**.953**	-.161	**.944**	-.019	**.875**	.023
5.Relaxed	**.848**	-.135	**.724**	-.027	**.827**	-.025	**.773**	-.008
6. Worn out	**.563**	.255	**.548**	.280	.408	**.498**	**.507**	.415
7. Happy with personal life	**.540**	.211	**.531**	.132	**.590**	.144	**.629**	.027
8. Restless	**.825**	.051	**.659**	.164	**.943**	-.094	**.863**	-.012
9. Shortness of breath	.255	**.542**	-.008	**.723**	.272	**.491**	**.424**	.386
10. Tearful	**.846**	-.260	**.635**	.117	**.916**	-.211	**.862**	-.183
11. More dependent	.397	**.417**	.197	**.675**	.140	**.706**	.130	**.726**
12. Social activities	.168	**.703**	**.490**	.448	.157	**.733**	-.041	**.891**
13. Others/less confidence in you	**.643**	.158	.157	**.512**	.039	**.637**	.017	**.705**
14. Chest pain	.221	**.560**	**.339**	.237	-.005	**.744**	-.053	**.795**
15. Lack self-confidence	**.671**	.256	**.618**	.270	**.732**	.168	**.619**	.292
16. Aching legs	**.399**	.275	.117	**.510**	.107	**.589**	.200	**.481**
17. Sports/exercise limited	-.224	**.946**	-.151	**.912**	-.034	**.789**	.165	**.605**
18. Frightened	**.709**	.146	**.789**	.011	**.689**	.167	**.574**	.285
19. Dizzy or lightheaded	**.520**	.125	**.613**	.067	.289	**.324**	**.570**	.071
20. Restricted or limited	-.101	**.923**	-.012	**.789**	-.021	**.844**	.097	**.787**
21. Unsure about exercise	-.001	**.796**	-.151	**.941**	-.136	**.842**	.092	**.649**
22. Overprotective family	-.200	.706	-.182	**.778**	-.162	**.663**	-.144	**.648**
23. Burden on others	**.460**	.307	.062	**.752**	-.190	**.940**	-.288	**.849**
24. Excluded	.123	**.649**	.346	**.448**	.198	**.632**	-.087	**.912**

25. Unable to socialize	.044	**.785**	**.472**	.434	.203	**.706**	.065	**.836**
26. Physical activity	-.223	**.985**	-.042	**.870**	-.001	**.879**	.254	**.686**
Variance explained	64.5%		65.1%		65.7%		65.7%	

Legend: Emo-emotional domain. Soc-social domain. Phys-physical domain. Values in bold represent the item factor loadings that group together in domains in the Serbian MacNew HRQL. Values in shade represent the distribution of items in the original MacNew HRQL.
